# Supplementary material for: Genome-Based Analysis of Virulence Factors and Biofilm Formation in Novel P. aeruginosa Strains Isolated from Household Appliances
Source: Microorganisms. 2022 Dec 19;10(12):2508. doi: 10.3390/microorganisms10122508 (PMC9781345; doi:10.3390/microorganisms10122508)
Supplement: Supplementary file 1 [file microorganisms-10-02508-s001.zip › microorganisms-2087541-supplementary.pdf]

Supplementary Material

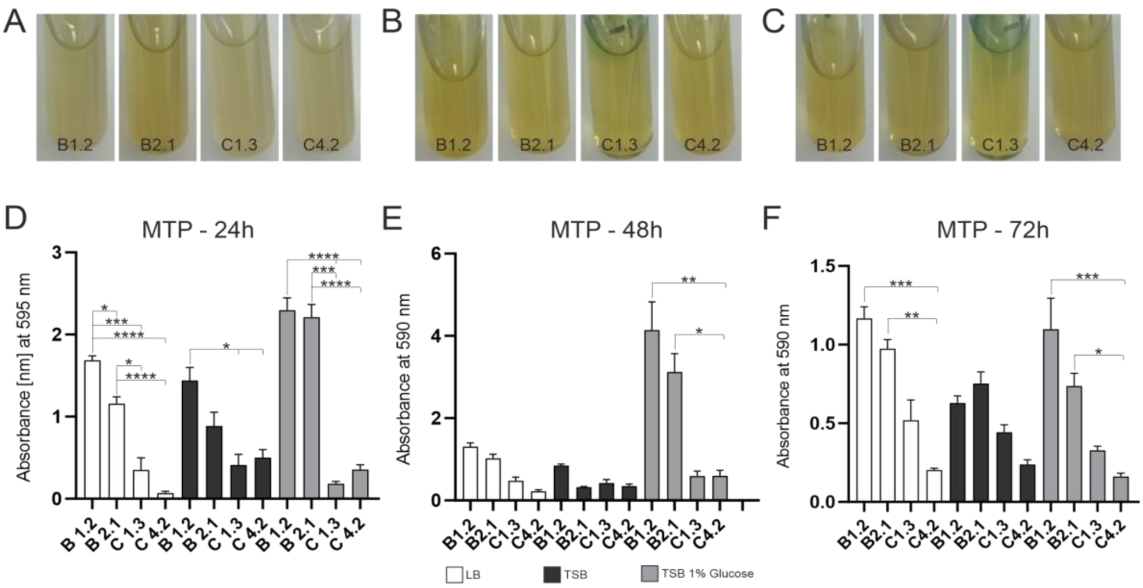

Figure S1. Investigation of Pseudomonas aeruginosa growth behavior and biofilm formation. P. aeruginosa isolates were incubated without agitation for 72h and pictures were taken every 24h (A: 24h, B: 48h, 72h). Additionally, biofilm formation was tested after 24h, 48h and 72h by the microtiter plate assay (D-F). Means ± SEM were statistically analyzed by Brown-Forsythe, Welch ANOVA tests (p < 0.05 (*), p < 0.01 (**), p < 0.001 (***), p < 0.0001 (****)), Data in D passed the D'Agostino & Pearson normality test (alpha=0.05). E and F did not passed D'Agostino and Pearson normality test, Means± SEM were were analyzed by Kuskal Wallis-test (p < 0.05 (*), p < 0.01 (**), p < 0.001 (***), p < 0.0001 (****)).

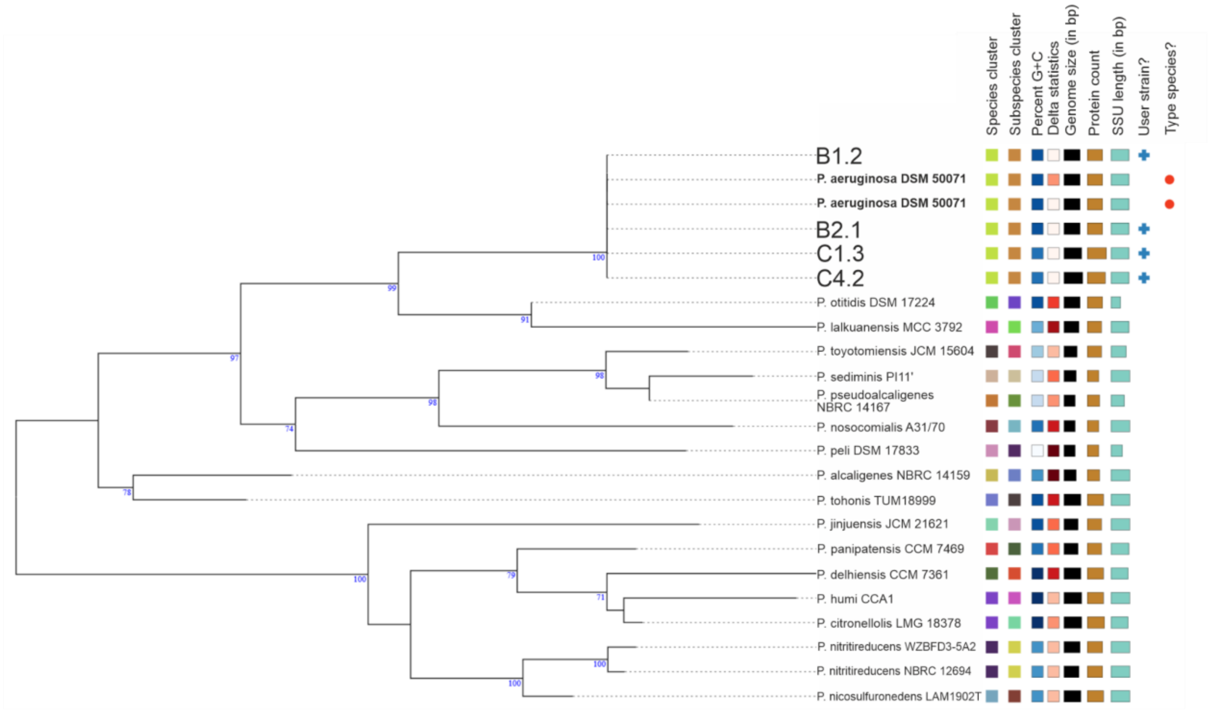

Figure S2. 16s rRNA tree. The 16S rRNA sequences were used for additional taxonomic classification of the strains. Phylogenetic 16S rRNA gene sequence tree and Genome-scale GBDP tree (Figure. 1) reveals that the four WM isolates belong to the species *P. aeruginosa*. Generated by using the type (strain) genome server (TYGS) [46,47].

Table S1. List of Prophages. Identified with PHASTER web tool.

Region	Region Length	Completeness	Score	# Total Proteins	Region Position	Most Common Phage	GC %
**B1.2**							
1	29.8Kb	incomplete	20	11	630129-660002	PHAGE_Klebsi_ST13_OXA48phi12.1_NC_049453(4)	65.14%
2	18.4Kb	questionable	80	24	679164-697633	PHAGE_Pseudo_phiCTX_NC_003278(6)	65.01%
3	54.9Kb	intact	120	68	2595731-2650671	PHAGE_Pseudo_phi297_NC_016762(42)	63.31%
4	18.4Kb	questionable	75	17	4273897-4292390	PHAGE_Pseudo_Pf1_NC_001331(7)	60.00%
5	11.6Kb	questionable	75	15	4782027-4793711	PHAGE_Pseudo_Pf1_NC_001331(7)	54.89%
6	13.2Kb	questionable	85	19	5307963-5321244	PHAGE_Pseudo_Pf1_NC_001331(8)	55.43%
7	46.2Kb	intact	137	56	5884615-5930832	PHAGE_Pseudo_Dobby_NC_048109(45)	63.27%
**B2.1**							
1	29.8Kb	incomplete	20	11	630129-660002	PHAGE_Klebsi_ST147_VIM1phi7.1_NC_049451(4)	65.14%
2	18.4Kb	questionable	80	24	679164-697633	PHAGE_Pseudo_Dobby_NC_048109(6)	65.01%
3	54.9Kb	intact	120	68	2595731-2650671	PHAGE_Pseudo_phi297_NC_016762(42)	63.31%
4	18.4Kb	questionable	75	17	4273884-4292377	PHAGE_Pseudo_Pf1_NC_001331(7)	60.00%
5	11.6Kb	questionable	75	15	4782014-4793698	PHAGE_Pseudo_Pf1_NC_001331(7)	54.89%
6	13.2Kb	questionable	85	19	5307950-5321231	PHAGE_Pseudo_Pf1_NC_001331(8)	55.43%
7	46.2Kb	intact	137	56	5884602-5930819	PHAGE_Pseudo_Dobby_NC_048109(45)	63.27%
**C1.3_1**							
1	17.2Kb	incomplete	40	7	614001-631219	PHAGE_Entero_fiAA91_ss_NC_022750(2)	62.98%

2	32.3Kb	intact	150	41	663492-695885	PHAGE_Pseudo_YMC11/02/R656_NC_028657(8)	64.01%
3	42.5Kb	intact	98	61	1695503-1738089	PHAGE_Pseudo_MD8_NC_031091(42)	61.66%
4	47.2Kb	intact	110	65	1881037-1928240	PHAGE_Pseudo_phi2_NC_030931(33)	62.66%
5	41.2Kb	questionable	80	54	3393486-3434699	PHAGE_Pseudo_JBD93_NC_030918(29)	64.93%
6	8.5Kb	incomplete	30	8	3759540-3768078	PHAGE_Escher_520873_NC_049344(2)	64.23%
7	11.3Kb	questionable	85	9	5382117-5393466	PHAGE_Pseudo_Pf1_NC_001331(8)	56.68%
8	12.1Kb	incomplete	30	19	5752075-5764220	PHAGE_Stx2_c_1717_NC_011357(2)	62.50%
**C1.3_2**							
1	6.9Kb	incomplete	30	10	138368-145290	PHAGE_Stenot_Moby_NC_048802(1)	57.24%
2	25.2Kb	incomplete	40	17	147690-172969	PHAGE_Pseudo_H66_NC_042342(3)	61.48%
3	9.3Kb	incomplete	40	13	167832-177160	PHAGE_Salmon_SJ46_NC_031129(2)	60.53%
**C1.3_3**							
1	32.4Kb	intact	150	51	2921-35389	PHAGE_Vibrio_vB_VpaM_MAR_NC_019722(13)	63.97%
**C4.2**							
**1**	16.1Kb	incomplete	20	12	684759-700920	PHAGE_Klebsi_ST147_VIM1phi7.1_NC_049451(4)	64.15%
**2**	18.4Kb	questionable	90	24	733539-752010	PHAGE_Pseudo_phiCTX_NC_003278(6)	64.96%
**3**	52.6Kb	intact	120	62	933322-985987	PHAGE_Pseudo_F10_NC_007805(37)	62.67%
**4**	38.8Kb	intact	109	56	1729615-1768453	PHAGE_Pseudo_JBD5_NC_020202(43)	64.43%
**5**	40.9Kb	intact	130	31	2464911-2505844	PHAGE_Stx2_c_Stx2a_F451_NC_049924(3)	61.31%

**6**	28.3Kb	intact	150	38	2555514-2583850	PHAGE_Escher_vB_EcoM_ECOO78_NC_041926(6)	63.28%
**7**	60.2Kb	intact	150	84	2796339-2856539	PHAGE_Pseudo_phi297_NC_016762(41)	61.81%
**8**	41.2Kb	intact	140	59	3264990-3306280	PHAGE_Pseudo_JD024_NC_024330(29)	64.71%
**9**	37.1Kb	intact	102	53	3720332-3757444	PHAGE_Pseudo_JBD88a_NC_020200(37)	64.35%
**10**	65.9Kb	intact	150	97	3914478-3980454	PHAGE_Pseudo_YMC11/02/R656_NC_028657(55)	59.66%
**11**	36.1Kb	intact	102	52	4047926-4084080	PHAGE_Pseudo_JBD88a_NC_020200(37)	64.29%
**12**	35.4Kb	incomplete	50	11	4483100-4518522	PHAGE_Escher_503458_NC_049341(2)	64.34%
**13**	7.3Kb	incomplete	60	9	5161326-5168642	PHAGE_Synech_Bellamy_NC_047838(3)	55.31%
**14**	27.2Kb	questionable	70	8	5360224-5387483	PHAGE_Escher_503458_NC_049341(2)	57.98%
**15**	11Kb	incomplete	40	14	5385270-5396289	PHAGE_Burkho_phi1026b_NC_005284(4)	58.85%
**16**	12.1Kb	incomplete	50	12	5589745-5601891	PHAGE_Strept_YDN12_NC_028974(1)	65.09%
**17**	28.4Kb	intact	100	18	6780196-6808606	PHAGE_Stx2_c_Stx2a_F451_NC_049924(3)	56.80%
**18**	13.7Kb	incomplete	60	10	6966057-6979809	PHAGE_Shigel_SfII_NC_021857(1)	58.90%
**19**	7.3Kb	incomplete	60	9	7299742-7307058	PHAGE_Synech_Bellamy_NC_047838(3)	55.31%

```
bci                     ATGTTGATCTTGACCCGCCGCCCCGGCGAAACCCTGCATATCGGCGACAACATCACCGTC    60
phi297_NC_016762.1      ATGCTGATCTTGACCCGCCGCCCCGGCGAAACCCTGCATATCGGCGACAACATCACCGTC    60
phi297_B1.2             ATGCTGATCCTCACCCGCCGAGTCGGCGAAACCCTGCATATCGGCGACAACATCACCATC    60
phi297_B2.1             ATGCTGATCCTCACCCGCCGAGTCGGCGAAACCCTGCATATCGGCGACAACATCACCATC    60
phi297_C4.2             ATGCTGATCCTGACCAGAAGACCCGGCGAAACCCTGCATATCGGCGACAACATCACCATC    60
                        *** ***** * ***.*..*.  **********************************.**

bci                     ACGGTCCTCGGCAGCCAGGGCGACCAGGTGCGCCTCGGCATCACCGCCCCGGACGACGTC    120
phi297_NC_016762.1      ACGGTCCTCAGCAGCCAAGGCGACCAGGTGCGCCTCGGCATCACCGCCCCGGACGACGTC    120
phi297_B1.2             ACGGTCCTCGGCAGCCAAGGCGACCAGGTGCGCCTCGGCATCACCGCCCCGGACGACGTC    120
phi297_B2.1             ACGGTCCTCGGCAGCCAAGGCGACCAGGTGCGCCTCGGCATCACCGCCCCGGACGACGTC    120
phi297_C4.2             ACGGTCCTCGGCAGCCAAGGCGACCAGGTGCGCCTCGGCATCACCGCCCCGGACGACGTC    120
                        *********.*******.******************************************

bci                     GCCATCCACCGCTCCGAGATCTACCAGCAGATCGGCAACGTCCGACCGGTGCCGCCGGCG    180
phi297_NC_016762.1      GCCATCCACCGCTCCGAGATCTACCAGCAGATTGGCAACGTCCGTCCGGTGCCGCCGGCG    180
phi297_B1.2             GCCATTCACCGCTCCGAGATCTACCAGCAGATCGGCAACGTCCGACCGGTGCCGCCGGCG    180
phi297_B2.1             GCCATTCACCGCTCCGAGATCTACCAGCAGATCGGCAACGTCCGACCGGTGCCGCCGGCG    180
phi297_C4.2             GCCATCCACCGCTCCGAGATCTACCAGCAGATCGGCAACGTCCGACCGGTGCCGCCGGCG    180
                        ***** ************************** ***********:***************

bci                     GAACTGGTCGAGGCCTGGAACCGCGAGCACCCGGCGCCAGCGCTGATCGAGTACCGCCCG    240
phi297_NC_016762.1      GAGCTGGTCGAGGCCTGGAACCGAGAGCACCCGGCGCCAGCGCTGATCGAATACCGGCCG    240
phi297_B1.2             GAACTGGTCGAAGCCTGGAACCGAGAGCACCCGGCGCCAGCGCTGATCGAATACCGGCCG    240
phi297_B2.1             GAACTGGTCGAAGCCTGGAACCGAGAGCACCCGGCGCCAGCGCTGATCGAATACCGGCCG    240
phi297_C4.2             GAACTGGTCGAGGCCTGGAACCGAGAGCACCCAGCGCCAGCGCTGATCGAGTACCGCCCG    240
                        **.********.***********.********.*****************.***** ***

bci                     TACCGAGGGGCCGAACCGCAGCGCACCCGCACCGTCGGCCGGGCCAGCGTGTCGCTTGGC    300
phi297_NC_016762.1      TACCGCGGGGCCGAACCGCAGCGCACCCGCACCGTCGGCCGGGCCAGCGTGTCGCTCGGC    300
phi297_B1.2             TACCGCGGGGCCGAACCGCAGCGCACCCGCACCGTCGGCCGGGCCAGCGTGTCGCTTGGC    300
phi297_B2.1             TACCGCGGGGCCGAACCGCAGCGCACCCGCACCGTCGGCCGGGCCAGCGTGTCGCTTGGC    300
phi297_C4.2             TACCGAGGGGCCGAACCGCAGCGCACCCGCACCGTCGGCCGGGCCAGCGTGTCGCTTGGC    300
                        *****.************************************************** ***

bci                     GGGGCGGCGGTTATCTGGATCGAAGGCCAGTCGGCGCCGGTGGCGTTGCGGGCCTGCACC    360
phi297_NC_016762.1      GGGGCCGCGGTTATCTGGATCGAAGGCCAATCGGCGCCGGTCGCGTTGCGGTCCTGCACT    360
phi297_B1.2             GGGGCGGCGGTTATCTGGATCGAAGGCCAGTCGGCGCCGGTGGCGTTGCGGGCCTGCGCC    360
phi297_B2.1             GGGGCGGCGGTTATCTGGATCGAAGGCCAGTCGGCGCCGGTGGCGTTGCGGGCCTGCGCC    360
phi297_C4.2             GGGGCCGCGGTTATCTGGATCGAAGGCCAGTCGGCGCCGGTGGCGTTGCGGGCCTGCACT    360
                        ***** ***********************.*********** ********* *****.* 

bci                     GCGGTCACCTGA    372
phi297_NC_016762.1      GCGATCTCCTGA    372
phi297_B1.2             GCGATCTCCTGA    372
phi297_B2.1             GCGATCTCCTGA    372
phi297_C4.2             GC----------    362
                        **
```

Figure S3. Multiple alignment of the *bci* gene sequence and the BLAST products. Gene sequence of the *bci* gene was taken from the AUS531phi phage [38].

```
bci                     MLILTRRPGETLHIGDNITVTVLGSQGDQVRLGITAPDDVAIHRSEIYQQIGNVRPVPPA    60
phi297_NC_016762.1      MLILTRRPGETLHIGDNITVTVLSSQGDQVRLGITAPDDVAIHRSEIYQQIGNVRPVPPA    60
phi297_B1.2             MLILTRRVGETLHIGDNITITVLGSQGDQVRLGITAPDDVAIHRSEIYQQIGNVRPVPPA    60
phi297_B2.1             MLILTRRVGETLHIGDNITITVLGSQGDQVRLGITAPDDVAIHRSEIYQQIGNVRPVPPA    60
phi297_C4.2             MLILTRRPGETLHIGDNITITVLGSQGDQVRLGITAPDDVAIHRSEIYQQIGNVRPVPPA    60
                        ******* ***********:***.************************************

bci                     ELVEAWNREHPAPALIEYRPYRGAEPQRTRTVGRASVSLGGAAVIWIEGQSAPVALRACT    120
phi297_NC_016762.1      ELVEAWNREHPAPALIEYRPYRGAEPQRTRTVGRASVSLGGAAVIWIEGQSAPVALRSCT    120
phi297_B1.2             ELVEAWNREHPAPALIEYRPYRGAEPQRTRTVGRASVSLGGAAVIWIEGQSAPVALRACA    120
phi297_B2.1             ELVEAWNREHPAPALIEYRPYRGAEPQRTRTVGRASVSLGGAAVIWIEGQSAPVALRACA    120
phi297_C4.2             ELVEAWNREHPAPALIEYRPYRGAEPQRTRTVGRASVSLGGAAVIWIEGQSAPVALRACT    120
                        *********************************************************:*:

bci                     AVT*    123
phi297_NC_016762.1      AIS*    123
phi297_B1.2             AIS*    123
phi297_B2.1             AIS*    123
phi297_C4.2             ----    120
```

Figure S4. Multiples alignment of the protein sequence of the *bci* gene. The *bci* protein sequence was taken from the AUS531phi phage [38].

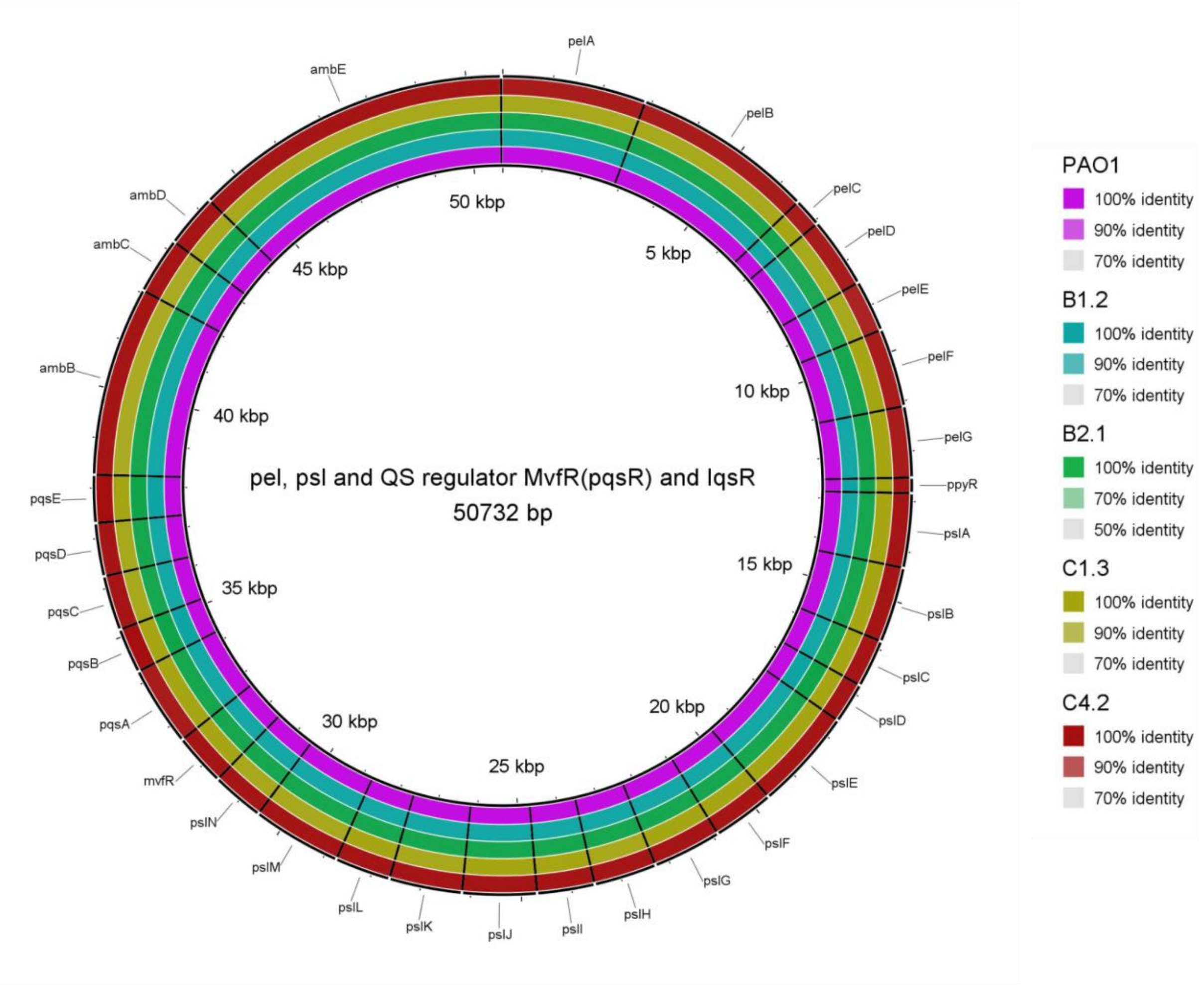

Figure S5. Presence and absence of the *pel, psl* locus, the psl operon pyoverdine operon regulator *ppyr,* the multiple virulence factor regulator (*MvfR also known as pqsR*, quorum sensing regulator) including the *pqs* operon and the *amb* operon belonging to the IqsR QS system. Blast Ring Image Generator (BRIG) was used for the multiple comparison of the presence and absence of the tested genes taken from the PAO1 reference strain against the washing machine isolates.

Table S2. List of virulence and biofilm-associated genes used for BRIG analysis. Gene sequences were downloaded from the Virulence factors of Pathogenic Bacteria DataBase (VFDB) and from NCBI. All Sequences were taken from the PAO1 reference strain except for the *exoU*, which is not present in PAO1, was taken from PA14.

Gene	Locus tag (PAO1)	Function
**Virulence factors of Pathogenic Bacteria DataBase (VFDB)**		
alg44	PA3542	Biofilm - Alginate (Mucoid exopolysaccharide)
alg8	PA3541	Biofilm - Alginate (Mucoid exopolysaccharide)
algA	PA3551	Biofilm - Alginate (Mucoid exopolysaccharide)
algC	PA5322	Biofilm - Alginate (Mucoid exopolysaccharide)
algD	PA3540	Biofilm - Alginate (Mucoid exopolysaccharide)
algE	PA3544	Biofilm - Alginate (Mucoid exopolysaccharide)

algF	PA3550	Biofilm - Alginate (Mucoid exopolysaccharide)
algG	PA3545	Biofilm - Alginate (Mucoid exopolysaccharide)
algI	PA3548	Biofilm - Alginate (Mucoid exopolysaccharide)
algJ	PA3549	Biofilm - Alginate (Mucoid exopolysaccharide)
algK	PA3543	Biofilm - Alginate (Mucoid exopolysaccharide)
algL	PA3547	Biofilm - Alginate (Mucoid exopolysaccharide)
algP/algR3	PA5253	Biofilm - Alginate (Mucoid exopolysaccharide)
algQ	PA5255	Biofilm - Alginate (Mucoid exopolysaccharide)
algR	PA5261	Biofilm - Alginate (Mucoid exopolysaccharide)
algU	PA0762	Biofilm - Alginate (Mucoid exopolysaccharide)
algW	PA4446	Biofilm - Alginate (Mucoid exopolysaccharide)
algX	PA3546	Biofilm - Alginate (Mucoid exopolysaccharide)
algZ	PA5262	Biofilm - Alginate (Mucoid exopolysaccharide)
aprA	PA1249	Exoenzyme - Alkaline protease
chpA	PA0413	Adherence - TypeIV pili
chpB	PA0414	Adherence - TypeIV pili
chpC	PA0415	Adherence - TypeIV pili
chpD	PA0416	Adherence - TypeIV pili
chpE	PA0417	Adherence - TypeIV pili
clpV1	PA0090	Effector delivery system - Hcp secretion island I (HSI-1)
clpV2	PA1662	Effector delivery system - Hcp secretion island II (HSI-2)
clpV3	PA2371	Effector delivery system - Hcp secretion island III (HSI-3)
dotU1	PA0078	Effector delivery system - Hcp secretion island I (HSI-1)
dotU2	PA1668	Effector delivery system - Hcp secretion island II (HSI-2)
dotU3	PA2362	Effector delivery system - Hcp secretion island III (HSI-3)
exoS	PA3841	Effector delivery system - Type III secretion system (TTSS) secreted effectors
exoT	PA0044	Effector delivery system - Type III secretion system (TTSS) secreted effectors
exoU	PA14_RS20960	Effector delivery system - Type III secretion system (TTSS) secreted effectors
exoY	PA2191	Effector delivery system - Type III secretion system (TTSS) secreted effectors
exsA	PA1713	Effector delivery system - Type III secretion system (TTSS)
exsB	PA1712	Effector delivery system - Type III secretion system (TTSS)
exsC	PA1710	Effector delivery system - Type III secretion system (TTSS)

exsD	PA1714	Effector delivery system - Type III secretion system (TTSS)
exsE	PA1711	Effector delivery system - Type III secretion system (TTSS)
fha1	PA0081	Effector delivery system - Hcp secretion island I (HSI-1)
fimT	PA4549	Adherence - TypeIV pili
fimU	PA4550	Adherence - TypeIV pili
fimV	PA3115	Adherence - TypeIV pili
fleI/flag	PA1093	Motility - Flagella
fleN	PA1454	Motility - Flagella
fleP/fliT	PA1096	Motility - Flagella
fleQ	PA1097	Motility - Flagella
fleR	PA1099	Motility - Flagella
fleS	PA1098	Motility - Flagella
flgA	PA3350	Motility - Flagella
flgB	PA1077	Motility - Flagella
flgC	PA1078	Motility - Flagella
flgD	PA1079	Motility - Flagella
flgE	PA1080	Motility - Flagella
flgF	PA1081	Motility - Flagella
flgG	PA1082	Motility - Flagella
flgH	PA1083	Motility - Flagella
flgI	PA1084	Motility - Flagella
flgJ	PA1085	Motility - Flagella
flgK	PA1086	Motility - Flagella
flgL	PA1087	Motility - Flagella
flgM	PA3351	Motility - Flagella
flgN	PA3352	Motility - Flagella
flhA	PA1452	Motility - Flagella
flhB	PA1449	Motility - Flagella
flhF	PA1453	Motility - Flagella
fliA	PA1455	Motility - Flagella
fliC	PA1092	Motility - Flagella
fliD	PA1094	Motility - Flagella

fliE	PA1100	Motility - Flagella
fliF	PA1101	Motility - Flagella
fliG	PA1102	Motility - Flagella
fliH	PA1103	Motility - Flagella
fliI	PA1104	Motility - Flagella
fliJ	PA1105	Motility - Flagella
fliK	PA1441	Motility - Flagella
fliL	PA1442	Motility - Flagella
fliM	PA1443	Motility - Flagella
fliN	PA1444	Motility - Flagella
fliO	PA1445	Motility - Flagella
fliP	PA1446	Motility - Flagella
fliQ	PA1447	Motility - Flagella
fliR	PA1448	Motility - Flagella
fliS	PA1095	Motility - Flagella
fptA	PA4221	Nutritional/Metabolic factor - Pyochelin
fpvA	PA2398	Nutritional/Metabolic factor - Pyoverdine
gacA	PA2586	Regulation - GacS/GacA two-component system
gacS	PA0928	Regulation - GacS/GacA two-component system
hcnA	PA2193	Antimicrobial activity/Competitive advantage - Hydrogen cyanide production
hcnB	PA2194	Antimicrobial activity/Competitive advantage - Hydrogen cyanide production
hcnC	PA2195	Antimicrobial activity/Competitive advantage - Hydrogen cyanide production
hcp1	PA0085	Effector delivery system - Hcp secretion island I (HSI-1)
hcp2	PA1512	Effector delivery system - Hcp secretion island II (HSI-2)
hcp3	PA2367	Effector delivery system - Hcp secretion island III (HSI-3)
hdtS	PA0005	Biofilm - Acylhomoserine lactone synthase
hisF2	PA3151	Biofilm - Acylhomoserine lactone synthase
hisH2	PA3152	Immune modulation - LPS
hsiA1	PA0082	Effector delivery system - Hcp secretion island I (HSI-1)
hsiA2	PA1656	Effector delivery system - Hcp secretion island II (HSI-2)
hsiA3	PA2360	Effector delivery system - Hcp secretion island III (HSI-3)
hsiB1/vipA/tssB	PA0083	Effector delivery system - Hcp secretion island I (HSI-1)

hsiB2	PA1657	Effector delivery system - Hcp secretion island II (HSI-2)
hsiB3	PA2365	Effector delivery system - Hcp secretion island III (HSI-3)
hsiC1/vipB/tssC	PA0084	Effector delivery system - Hcp secretion island I (HSI-1)
hsiC2	PA1658	Effector delivery system - Hcp secretion island II (HSI-2)
hsiC3	PA2366	Effector delivery system - Hcp secretion island III (HSI-3)
hsiE1	PA0086	Effector delivery system - Hcp secretion island I (HSI-1)
hsiF1/tssE	PA0087	Effector delivery system - Hcp secretion island I (HSI-1)
hsiF2	PA1659	Effector delivery system - Hcp secretion island II (HSI-2)
hsiF3	PA2368	Effector delivery system - Hcp secretion island III (HSI-3
hsiG1/tssf	PA0088	Effector delivery system - Hcp secretion island I (HSI-1)
hsiG2	PA1660	Effector delivery system - Hcp secretion island II (HSI-2)
hsiG3	PA2369	Effector delivery system - Hcp secretion island III (HSI-3)
hsiH1	PA0089	Effector delivery system - Hcp secretion island I (HSI-1)
hsiH2	PA1661	Effector delivery system - Hcp secretion island II (HSI-2)
hsiH3	PA2370	Effector delivery system - Hcp secretion island III (HSI-3)
hsiJ1	PA0079	Effector delivery system - Hcp secretion island I (HSI-1)
hsiJ2	PA1667	Effector delivery system - Hcp secretion island II (HSI-2
hsiJ3	PA2363	Effector delivery system - Hcp secretion island III (HSI-3)
icmF1/tssM1	PA0077	Effector delivery system - Hcp secretion island I (HSI-1)
icmF2	PA1669	Effector delivery system - Hcp secretion island II (HSI-2)
imcF3	PA2361	Effector delivery system - Hcp secretion island III (HSI-3)
lasA	PA1871	Effector delivery system - LasA (Elastase)
lasB	PA3724	Effector delivery system - LasB (Elastase)
lasI	PA1432	Biofilm - Quorum sensing
lasR	PA1430	Biofilm - Quorum sensing
lip1	PA0080	Effector delivery system - Hcp secretion island I (HSI-1)
lip2	PA1666	Effector delivery system - Hcp secretion island II (HSI-2)
motA	PA4954	Motility - Flagella
motB	PA4953	Motility - Flagella
motC	PA1460	Motility - Flagella
motD	PA1461	Motility - Flagella
motY	PA3526	Motility - Flagella

mucA	PA0763	Biofilm - Alginate (Mucoid exopolysaccharide)
mucB	PA0764	Biofilm - Alginate (Mucoid exopolysaccharide)
mucC	PA0765	Biofilm - Alginate (Mucoid exopolysaccharide)
mucD	PA0766	Biofilm - Alginate (Mucoid exopolysaccharide)
mucE	PA4033	Biofilm - Alginate (Mucoid exopolysaccharide)
mucP	PA3649	Biofilm - Alginate (Mucoid exopolysaccharide)
PA3142	PA3142	Immune modulation - LPS
PA3143	PA3143	Immune modulation - LPS
PA3157	PA3157	Immune modulation - LPS
pchA	PA4231	Nutritional/Metabolic factor - Pyochelin
pchB	PA4230	Nutritional/Metabolic factor - Pyochelin
pchC	PA4229	Nutritional/Metabolic factor - Pyochelin
pchD	PA4228	Nutritional/Metabolic factor - Pyochelin
pchE	PA4226	Nutritional/Metabolic factor - Pyochelin
pchF	PA4225	Nutritional/Metabolic factor - Pyochelin
pchG	PA4224	Nutritional/Metabolic factor - Pyochelin
pchH	PA4223	Nutritional/Metabolic factor - Pyochelin
pchI	PA4222	Nutritional/Metabolic factor - Pyochelin
pchR	PA4227	Nutritional/Metabolic factor - Pyochelin
pcr1	PA1699	Effector delivery system - Type III secretion system (TTSS)
pcr2	PA1700	Effector delivery system - Type III secretion system (TTSS)
pcr3	PA1701	Effector delivery system - Type III secretion system (TTSS)
pcr4	PA1702	Effector delivery system - Type III secretion system (TTSS)
pcrD	PA1703	Effector delivery system - Type III secretion system (TTSS)
pcrG	PA1705	Effector delivery system - Type III secretion system (TTSS)
pcrH	PA1707	Effector delivery system - Type III secretion system (TTSS)
pcrR	PA1704	Effector delivery system - Type III secretion system (TTSS)
pcrV	PA1706	Effector delivery system - Type III secretion system (TTSS)
phzA1	PA4210	Nutritional/Metabolic factor - Pyocyanin
phzA2	PA1899	Nutritional/Metabolic factor - Pyocyanin
phzB1	PA4211	Nutritional/Metabolic factor - Pyocyanin
phzB2	PA1900	Nutritional/Metabolic factor - Pyocyanin

phzC1	PA4212	Nutritional/Metabolic factor - Pyocyanin
phzC2	PA1901	Nutritional/Metabolic factor - Pyocyanin
phzD1	PA4213	Nutritional/Metabolic factor - Pyocyanin
phzD2	PA1902	Nutritional/Metabolic factor - Pyocyanin
phzE1	PA4214	Nutritional/Metabolic factor - Pyocyanin
phzE2	PA1903	Nutritional/Metabolic factor - Pyocyanin
phzF1	PA4215	Nutritional/Metabolic factor - Pyocyanin
phzF2	PA1904	Nutritional/Metabolic factor - Pyocyanin
phzG1	PA4216	Nutritional/Metabolic factor - Pyocyanin
phzG2	PA1905	Nutritional/Metabolic factor - Pyocyanin
phzH	PA0051	Nutritional/Metabolic factor - Pyocyanin
phzM	PA4209	Nutritional/Metabolic factor - Pyocyanin
phzS	PA4217	Nutritional/Metabolic factor - Pyocyanin
pilA	PA4525	Adherence - TypeIV pili
pilB	PA4526	Adherence - TypeIV pili
pilC	PA4527*	Adherence - TypeIV pili
pilE	PA4556	Adherence - TypeIV pili
pilF	PA3805	Adherence - TypeIV pili
pilG	PA0408	Adherence - TypeIV pili
pilH	PA0409	Adherence - TypeIV pili
pilI	PA0410	Adherence - TypeIV pili
pilJ	PA0411	Adherence - TypeIV pili
pilK	PA0412	Adherence - TypeIV pili
pilM	PA5044	Adherence - TypeIV pili
pilN	PA5043	Adherence - TypeIV pili
pilO	PA5042	Adherence - TypeIV pili
pilP	PA5041	Adherence - TypeIV pili
pilQ	PA5040	Adherence - TypeIV pili
pilR	PA4547	Adherence - TypeIV pili
pilS	PA4546	Adherence - TypeIV pili
pilT	PA0395	Adherence - TypeIV pili
pilU	PA0396	Adherence - TypeIV pili

pilV	PA4551	Adherence - TypeIV pili
pilW	PA4552	Adherence - TypeIV pili
pilX	PA4553	Adherence - TypeIV pili
pilY1	PA4554	Adherence - TypeIV pili
pilY2	PA4555	Adherence - TypeIV pili
pilZ	PA2960	Adherence - TypeIV pili
plcB	PA0026	Exotoxin - Phospholipase X
plcH	PA0844	Exotoxin - Phospholipase C
plcN	PA3319	Exotoxin - Non-hemolytic phospholipase C
pldA/tle5a	PA3487	Effector delivery system - HSI-2 T6SS secreted effectors
popB	PA1708	Effector delivery system - Type III secretion system (TTSS)
popD	PA1709	Effector delivery system - Type III secretion system (TTSS)
popN	PA1698	Effector delivery system - Type III secretion system (TTSS)
ppkA	PA0074	Effector delivery system - Hcp secretion island I (HSI-1)
pppA	PA0075	Effector delivery system - Hcp secretion island I (HSI-1)
prpL	PA4175	Exoenzyme - Protease IV
pscB	PA1715	Type III secretion system (TTSS)
pscC	PA1716	Type III secretion system (TTSS)
pscD	PA1717	Type III secretion system (TTSS)
pscE	PA1718	Type III secretion system (TTSS)
pscF	PA1719	Type III secretion system (TTSS)
pscG	PA1720	Type III secretion system (TTSS)
pscH	PA1721	Type III secretion system (TTSS)
pscI	PA1722	Type III secretion system (TTSS)
pscJ	PA1723	Type III secretion system (TTSS)
pscK	PA1724	Type III secretion system (TTSS)
pscL	PA1725	Type III secretion system (TTSS)
pscN	PA1697	Type III secretion system (TTSS)
pscO	PA1696	Type III secretion system (TTSS)
pscP	PA1695	Type III secretion system (TTSS)
pscQ	PA1694	Type III secretion system (TTSS)
pscR	PA1693	Type III secretion system (TTSS)

pscS	PA1692	Type III secretion system (TTSS)
pscT	PA1691	Type III secretion system (TTSS)
pscU	PA1690	Type III secretion system (TTSS)
pvdA	PA2386	Nutritional/Metabolic factor - Pyoverdine
pvdD	PA2399	Nutritional/Metabolic factor - Pyoverdine
pvdE	PA2397	Nutritional/Metabolic factor - Pyoverdine
pvdF	PA2396	Nutritional/Metabolic factor - Pyoverdine
pvdG	PA2425	Nutritional/Metabolic factor - Pyoverdine
pvdH	PA2413	Nutritional/Metabolic factor - Pyoverdine
pvdI	PA2402	Nutritional/Metabolic factor - Pyoverdine
pvdJ	PA2400	Nutritional/Metabolic factor - Pyoverdine
pvdL	PA2424	Nutritional/Metabolic factor - Pyoverdine
pvdM	PA2393	Nutritional/Metabolic factor - Pyoverdine
pvdN	PA2394	Nutritional/Metabolic factor - Pyoverdine
pvdO	PA2395	Nutritional/Metabolic factor - Pyoverdine
pvdP	PA2392	Nutritional/Metabolic factor - Pyoverdine
pvdQ	PA2385	Nutritional/Metabolic factor - Pyoverdine
pvdS	PA2426	Nutritional/Metabolic factor - Pyoverdine
pvdY	PA2427	Nutritional/Metabolic factor - Pyoverdine
rhlA	PA3479	Immune modulation - Rhamnolipid
rhlB	PA3478	Immune modulation - Rhamnolipid
rhlC	PA1130	Immune modulation - Rhamnolipid
rhlI	PA3476	Biofilm - Quorum sensing
rhlR	PA3477	Biofilm - Quorum sensing
tagF/pppB	PA0076	Effector delivery system - Hcp secretion island I (HSI-1)
tagR	PA0071	Effector delivery system - Hcp secretion island I (HSI-1)
tagS	PA0072	Effector delivery system - Hcp secretion island I (HSI-1)
tagT	PA0073	Effector delivery system - Hcp secretion island I (HSI-1)
toxA	PA1148	Exotoxin - Exotoxin A
tse1	PA1844	Effector delivery system - Hcp secretion island I (HSI-1) - T6SS secreted effectors
tse2	PA2702	Effector delivery system - Hcp secretion island I (HSI-1) - T6SS secreted effectors
tse3	PA3484	Effector delivery system - Hcp secretion island I (HSI-1) - T6SS secreted effectors

vgrG1a	PA0091	Effector delivery system - Hcp secretion island I (HSI-1)
vgrG2	PA1511	Effector delivery system - Hcp secretion island II (HSI-2)
vgrG3	PA2373	Effector delivery system - Hcp secretion island III (HSI-3)
waaA	PA4988	Immune modulation - LPS
waaC	PA5011	Immune modulation - LPS
waaF	PA5012	Immune modulation - LPS
waaG	PA5010	Immune modulation - LPS
waaP	PA5009	Immune modulation - LPS
wbpA	PA3159	Immune modulation - LPS
wbpB	PA3158	Immune modulation - LPS
wbpD	PA3156	Immune modulation - LPS
wbpE	PA3155	Immune modulation - LPS
wbpG	PA3150	Immune modulation - LPS
wbpH	PA3149	Immune modulation - LPS
wbpI	PA3148	Immune modulation - LPS
wbpJ	PA3147	Immune modulation - LPS
wbpK	PA3146	Immune modulation - LPS
wbpL	PA3145	Immune modulation - LPS
wbpM	PA3141	Immune modulation - LPS
wzx	PA3153	Immune modulation - LPS
wzy	PA3154	Immune modulation - LPS
wzz	PA3160	Immune modulation - LPS
xcpA/pilD	PA4528	Adherence - TypeIV pili
**NCBI**		
pelA	PA3064	Biofilm - hypothetical protein
pelB	PA3063	Biofilm - pellicle/biofilm biosynthesis protein PelB
pelC	PA3062	Biofilm - pellicle/biofilm biosynthesis outer membrane protein PelC
pelD	PA3061	Biofilm - pellicle/biofilm biosynthesis protein PelD
pelE	PA3060	Biofilm - pellicle/biofilm biosynthesis protein PelE
pelF	PA3059	Biofilm - pellicle/biofilm biosynthesis glycosyltransferase PelF
pelG	PA3058	Biofilm - pellicle/biofilm biosynthesis Wzx-like polysaccharide transporter PelG
ppyR	PA2663	Biofilm - psl and pyoverdine operon regulator PpyR

pslA	PA2231	Biofilm - biofilm formation protein PslA
pslB	PA2232	Biofilm - biofilm formation protein PslB
pslC	PA2233	Biofilm - biofilm formation protein PslC
pslD	PA2234	Biofilm - biofilm formation protein PslD
pslE	PA2235	Biofilm - biofilm formation protein PslE
pslF	PA2236	Biofilm - biofilm formation protein PslF
pslG	PA2237	Biofilm - biofilm formation protein PslG
pslH	PA2238	Biofilm - biofilm formation protein PslH
pslI	PA2239	Biofilm - biofilm formation protein PslI
pslJ	PA2240	Biofilm - biofilm formation protein PslJ
pslK	PA2241	Biofilm - biofilm formation protein PslL
pslL	PA2242	Biofilm - hypothetical protein
pslM	PA2243	Biofilm - FAD-binding dehydrogenase
pslN	PA2244	Biofilm - hypothetical protein
mvfR	PA1003	Quorum sensing - transcriptional regulator MvfR
pqsA	PA0996	Quorum sensing - anthranilate--CoA ligase
pqsB	PA0997	Quorum sensing - hypothetical protein
pqsC	PA0998	Quorum sensing - hypothetical protein
pqsD	PA0999	Quorum sensing - 3-oxoacyl-ACP synthase
pqsE	PA1000	Quorum sensing - thioesterase PqsE
